# PRELIMINARY SOMMA DATA: D3CR MUSCLE MASS AND STRENGTH AND WALKING SPEED IN OLDER MEN AND WOMEN

**DOI:** 10.1093/geroni/igac059.835

**Published:** 2022-12-20

**Authors:** Peggy Cawthon, Stephen Kritchevsky, Anne Newman, Theresa Mau, Russell T Hepple, Paul Coen, Bret Goodpaster, Steven Cummings

**Affiliations:** California Pacific Medical Center Research Institute, San Francisco, California, United States; Wake Forest School of Medicine, Winston-Salem, North Carolina, United States; University of Pittsburgh, Pittsburgh, Pennsylvania, United States; California Pacific Medical Center, San Francisco, California, United States; University of Florida, Gainesville, Florida, United States; AdventHealth, Orlando, Florida, United States; AdventHealth, Orlando, Florida, United States; San Francisco Coordinating Center, San Francisco, California, United States

## Abstract

D3Cr muscle mass, grip strength, leg extension 1-rm max (Keiser), and walking speed (400m usual walk) were collected with standardized protocols. We calculated sex-stratified unadjusted Pearson correlation coefficients, and partial Pearson correlation coefficient adjusted for body size (height, weight). D3Cr muscle mass was positively correlated with grip strength (men: unadjusted r=0.28, p<.001; women: unadjusted r=0.16, p=0.02) and leg extension strength (men: unadjusted r=0.44, p<.001; women: unadjusted r=0.31, p<.001). D3Cr muscle mass was correlated with 400-m walk speed only after adjustment for body size among both men (partial r=0.29, p<.001) and women (partial r=0.16, p=0.02). Associations of total thigh muscle volume by MRI with strength and walking speed were of similar magnitude to the association between D3Cr muscle mass with strength and walking speed.

